# Perceptions in orthopedic surgery on the use of cannabis in treating pain: a survey of patients with spine pain (POSIT Spine)

**DOI:** 10.1186/s13018-024-04558-6

**Published:** 2024-01-30

**Authors:** Marko Gjorgjievski, Kim Madden, Conner Bullen, Frank Koziarz, Alex Koziarz, Aleksa Cenic, Silvia Li, Mohit Bhandari, Herman Johal

**Affiliations:** 1 Division of Orthopedic Surgery, Department of Surgery, Victory 3, Kingston General Hospital, Queen’s University, 76 Stuart Street, Kingston, ON K7L 2V7 Canada; 2 Department of Health Research Methods, Evidence and Impact, McMaster University, Hamilton, ON Canada; 3 Division of Orthopedic Surgery, Department of Surgery, McMaster University, Hamilton, ON Canada; 4 Faculty of Pharmacy, University of Toronto, Toronto, Canada; 5 Department of Radiology, McMaster University, Hamilton, ON Canada; 6 Division of Neurosurgery, Department of Surgery, McMaster, University, Hamilton, Canada

**Keywords:** Cannabis, Spine pain, Back pain, MSK pain, Orthopedics, Opioids, Opioid epidemic, Opioid alternative, Patient perceptions, Survey

## Abstract

**Background:**

Back pain is the leading cause of disability worldwide. Despite guidelines discouraging opioids as first-line treatment, opioids remain the most prescribed drugs for back pain. There is renewed interest in exploring the potential medical applications of cannabis, and with the recent changes in national legislation there is a unique opportunity to investigate the analgesic properties of cannabis.

**Methods:**

This was a multi-center survey-based study examining patient perceptions regarding cannabis for spine pain. We included patients presenting with back or neck pain to one of three Orthopedic clinics in Ontario. Our primary outcome was perceived effect of cannabis on back pain, while secondary outcomes were perceptions regarding potential applications and barriers to cannabis use.

**Results:**

259 patients participated in this study, 35.3% (90/255) stating they used cannabis medically. Average pain severity was 6.5/10 ± 0.3 (95% CI 6.2–6.8). Nearly three-quarters were prescribed opioids (73.6%, 148/201), with oxycodone/oxycontin (45.9% 68/148) being the most common, and almost half of (49.3%, 73/148) had used an opioid in the last week. Patients estimated cannabis could treat 54.3% ± 4.0 (95% CI 50.3–58.3%) of their spine pain and replace 46.2% ± 6. 6 (95% CI 39.6–52.8%) of their current analgesics. Age (*β* = − 0.3, CI − 0.6–0.0), higher pain severity (*β* = 0.4, CI 0.1–0.6) and previous cannabis use (*β* = 14.7, CI 5.1–24.4) were associated with a higher perceived effect of cannabis. Patients thought cannabis would be beneficial to treat pain (129/146, 88.4%), and reduce (116/146, 79.5%) or eliminate opioids (102/146, 69.9%). Not considering using cannabis for medical purposes (65/150, 43.3%) was the number one reported barrier.

**Conclusions:**

Patients estimated medical cannabis could treat more than half of their spine pain, with one in three patients already using medical cannabis. 79% of patients also believe cannabis could reduce opioid usage. This data will help support more research into cannabis for musculoskeletal pain.

## Background

Back pain is the leading cause of activity limitation and work absence worldwide, with back-related disability and population burden on the rise [1]. It affects 50–80% of the population [2]. Furthermore, it is associated with an enormous economic burden on individuals, families, communities, industry, and governments [3, 4]. In the United States (US) alone, the annual costs of back pain exceed US$100 billion [5].

Treatment guidelines for non-specific back pain recommend non-pharmacological care as first-line and discourage the use of opioids [6, 7]. Pharmacological treatment should only follow if there is an inadequate response to non-pharmacological interventions, and then it should start with oral NSAIDs at the lowest effective dose for the shortest possible time [8]. Opioids, although effective for treating pain, come with significant risks and potential side effects, including addiction, tolerance, and overdose, and therefore their routine use is not recommended and should only be used for a short duration in carefully selected patients, and with appropriate monitoring [9].

However, despite a lack of high-quality data on the benefits, opioids are still commonly prescribed for chronic musculoskeletal (MSK) pain [10]. Insurance claims data suggest that opioids are the most commonly prescribed class of drug for back pain [11]. This problem seems to be characteristic of the US and Canada, which prescribe postoperative opioids in higher doses and more frequently than other countries [12, 13]. One-fifth of all opioid-related deaths are linked to prescriptions [14], and orthopedic surgeons are the third-highest prescribers of opioids [15]. In 2016, there were approximately 3000 apparent opioid-related deaths in Canada, which is equivalent to eight people dying every day [16]. This number has more than doubled in 2021 (7169) [17]. The restrictions implemented to control the COVID-19 spread have also limited the access to services essential for vulnerable populations, resulting in a higher risk of withdrawal, overdose, and death [18, 19].

North America has been going through an opioid epidemic, and opioid-sparing alternatives are needed for pain management. There are many recorded instances of cannabis being used as an analgesic throughout human history, some as early as the second century AD [20]. Cannabis contains cannabinoids, which have been found to have pain-relieving and anti-inflammatory properties. In recent years, there has been growing interest in cannabis for pain management, including for back and neck pain. Nevertheless, high-quality data regarding cannabis use for pain management in Orthopedic surgery is scarce, indicating that more research is needed [21, 22]. While research on the effectiveness of cannabis for pain management is still ongoing, some have reported significant improvements in their symptoms with its use [23]. In Canada, medical cannabis has been legal since 2001, and in 2018 Canada legalized the recreational use of cannabis, presenting a unique setting for cannabis research.

The objectives of this study were to examine the patients' positions and perceptions regarding cannabis in the treatment of back pain. The primary aim was to determine the perceived effects of cannabis on back and neck pain. Our secondary goals were to explore patient preferences, insight, and attitudes surrounding cannabis use for MSK pain management and identify possibilities for future research.

## Methods

This is a multi-center survey-based study designed to determine patients' positions, preferences, and insight on cannabis use for back pain. English-speaking adult patients presenting at the three participating clinics (Hamilton, ON) with back or neck pain were screened for inclusion. Patients who were cognitively impaired, too ill or injured to participate, or being at the clinics for a traumatic injury or arthritis were excluded.

The questionnaire was developed by consulting current literature, patients, and a multidisciplinary group of experts (orthopedic surgeons, rheumatologists, anesthesiologists, epidemiologists, cannabis dispensary operators and licensed producers). It consisted of 50 questions using a multiple-choice and Likert-scale format to enhance the answer rate [24]. The questionnaire collected information on patient and injury characteristics; pain severity and analgesic usage; perceptions and positions about medical cannabis; perceived effectiveness of medical cannabis for back pain; and barriers to appropriate clinical use. We used the visual analog scale (VAS) to estimate pain severity, starting from 0 for no pain to 100 for severe pain. We used a continuous scale (0–100%) to measure the perceived effectiveness of cannabis, where patients rated how much pain they felt could be or is being treated by cannabis. We also assessed patients for anxiety and post-traumatic stress disorder (PTSD) using the PROMIS (Patient-Reported Outcomes Measurement Information System) SF v1.0-Anxiety 4a (four questions) and the Short Screening Scale version of the DSM-IV PTSD test (seven questions) [25, 26]. We then tested the questionnaire draft through interviews and focused feedback with our group of experts for comprehensiveness, readability, and clarity.

Members of the research team approached patients consecutively at the participating clinics and screened them for study inclusion. Eligible patients that provided consent completed the questionnaire administered by the research member using a tablet device. Surveys were anonymous, and the anonymous data was entered into the REDCap database. We summarized continuous data as means and standard deviations and categorical data as counts and percentages. Our primary outcome was perceived effect of cannabis on back pain, while secondary outcomes were perceptions regarding potential applications and barriers to cannabis use. Additionally, a regression analysis to determine associations with patients' perceived effectiveness of cannabis was done. The independent variables were patient and injury characteristics, previous surgery for the pain, pain frequency and severity, opioid use, past experience with cannabis, and the presence of PTSD and anxiety. The P-values were two-tailed, with a threshold of 0.05 considered statistically significant.

We calculated our sample size based on our primary regression analysis. We used a sample size calculation with a significance level 0.05 with an estimated medium effect size of 0.3 to be powered for 80%. This was a conservative estimate. Accounting for possible correlation and response categories, an estimated 174 patients would achieve adequate power to assess the relationship for perceived effectiveness. We also added an additional 20% to account for patients with incomplete data.

## Results

### Patient and injury characteristics

Data collection was done from January 24, 2018, to March 7, 2018. There were 259 patients presenting with neck and back pain that participated in this study. The participants' mean age was 53.9 years old (range 19–99). Of all the participants, 50.2% (123/245) identified as male, and 43.3% (106/245) as female. The patient demographics and injury characteristics are presented in Tables 1 and 2, respectively. Most patients had back pain for longer than 6 months (228/255, 89.4%) versus less than 6 months (27/255, 10.6%). However, 88.8% (230/259) of patients experienced pain within the last week, with 121 patients (46.7%) having had surgery for their spine pain. The mean reported VAS was 6.5/10 ± 0.3 (95% CI 6.2–6.8) (Table 2). The average VAS score for cannabis users was 6.8 ± 0.4 (95% CI 6.5–7.2, *P* < 0.001) and 6.5 ± 0.5 (95% CI 6.0–6.9, *P* < 0.001) for non-cannabis users. Additionally, 32.2 (57/177) and 30.0% (54/180) of patients screened positive for PTSD and anxiety, respectively.

**Table 1:** Patient demographics

Variable	No. of patients (%)
Total number of participants	259 (100)
Age (*N* = 239)	Mean (SD) 53.9 (15.4)
Sex (*N* = 245)	
Female	106 (40.3)
Male	123 (50.2)
*Prefer not to answer*	16 (6.5)
*Education (N = 256)*	
Less than high school	18 (7)
High school	88 (34.4)
College/ trade school	102 (39.8)
Undergraduate degree	35 (13.7)
Graduate degree	13 (5.1)
*Income (N = 213)*	
< $25,000	61 (28.6)
$25,000–$49,999	51 (23.9)
$50,000–$74,999	41 (19.2)
$74,999–$99,999	33 (15.5)
> $99,999	27 (12.7)

**Table 2:** Injury characteristics

Variable	No. of patients (%)
Duration of symptoms	255 (100)
*Less than 6 months (acute)*	27 (10.6)
*6 months or more (chronic)*	228 (89.4)
Underwent surgery for musculoskeletal injury	256 (100)
*No*	138 (53.3)
*Yes,*	121 (46.7)
*Within the last month*	30 (24.8%)
*In the last 1–12 months*	45 (37.2%)
*Over 1 year ago*	45 (37.2%)
*Unspecified*	1 (0.01)
Experienced musculoskeletal pain in the past week	259 (100)
*Yes*	230 (88.8)
*No*	21 (8.1)
*Unsure*	8 (3.1)
Pain severity (VAS) (*N* = 242)	Mean (SD) 6.5 (2.4)
*No pain (VAS 0)*	4 (1.7%)
*Minimal pain (VAS 1–3)*	23 (9.5%)
*Moderate pain (VAS 4–6)*	61 (25.5%)
*Severe pain (VAS 7–9)*	125 (51.7%)
*Extreme pain (VAS 10)*	29 (12.0%)
Pain severity (VAS) for cannabis users (*N* = 135)	Mean (SD) 6.8 (2.2)*
Pain severity (VAS) for non-cannabis users (*N* = 105)	Mean (SD) 6.5 (2.6)*

VAS = Visual analog scale

*p-value < *0.05*

### Analgesic use

There were 208 (80.9%, 208/257) patients that were prescribed an analgesic (Table 3). Of all patients taking analgesics, nearly three-quarters were prescribed opioids (73.6%, 148/201), with oxycodone/oxycontin (45.9% 68/148) being the most common and fentanyl as the least (4.1%, 6/148). Almost half of the patients (49.3%, 73/148) had used an opioid in the last week. Over-the-counter medications were also prescribed (77.1%, 199/258), with 69.4% (136/196) of patients using ibuprofen and 64.3% (126/196) using acetaminophen. Additionally, 46.3% (93/201) of patients used other NSAIDs. Of these, naproxen was the most common (50.5%, 47/93). Regarding cannabis, over a third of the patients (35.3%, 90/255) stated they used cannabis medicinally, with 31.8% (81/255) having used it in the previous year specifically for their pain (Table 3). Additionally, 40% (102/253) reported using cannabis recreationally.

**Table 3:** Analgesic use

Variable	No. of patients (%)	
*Use of prescription analgesics for current musculoskeletal pain (N = 257)*		
*Yes*	208 (80.9)	
*No*	49 (19.1)	
Prescription medications	Prescribed for MSK pain (*n* = 201)	
*Opioids*	148 (73.6%)	
*Oxycodone/Oxycontin*	70 (47.3%)	
*Codeine*	59 (39.9%)	
*Hydromorphone*	50 (33.8%)	
*Morphine (oral)*	24 (16.2%)	
*Fentanyl (oral/patch)*	6 (4.1%)	
*Opioids used in last week*	73 (49.3%)	
*NSAIDs*	93 (46.3%)	
*Naproxen*	47 (50.5%)	
*Celecoxib*	31 (33.3%)	
*Toradol*	24 (25.8%)	
*Diclofenac (topical)*	12 (12.9%)	
*Meloxicam*	7 (7.5%)	
*Diclofenac (oral)*	6 (6.5%)	
*Gabapentin/Pregabalin*	*75 (37.3%)*	
*Use non-prescription/OTC analgesics for current musculoskeletal pain (N = 258)*		
*No*	59 (28.3%)	
*Yes*	199 (77.1%)	
*Ibuprofen*	136 (69.4%)	
*Acetaminophen*	126 (64.3%)	
*Naproxen*	67 (34.2%)	
*Acetylsalicylic Acid*	35 (17.9%)	
*Diclofenac (topical)*	35 (17.9%)	
Used cannabis, or know someone who has	Medically (*N* = 255)	Recreationally (*N* = 253)
*No*	100 (39.2%)	93 (36.8%)
*Yes*	155 (60.8%)	160 (63.2%)
*Used and know someone*	48 (18.8%)	67 (26.5%)
*Used*	42 (16.5%)	35 (13.8%)
*Know someone*	65 (25.5%)	58 (22.9%)
*Used cannabis in the past 12 months to control pain (N = 255)*		
*No*	174 (68.2%)	
*Yes*	81 (31.8%)	

*N* No. = Number, *MSK* Musculoskeletal, *NSAIDs* Non-Steroidal Anti-inflammatory Drugs

*Where data were missing or a study participant did not respond to a query, percentages were calculated out of the total number of responses, not the number of study patients

**Muscle relaxants, balms/rubs/cream, herbal medications (i.e.,, Arnica, Turmeric)

### Perceived effects of cannabis on musculoskeletal pain

Patients believed that cannabis could treat 54.3% ± 4.0 (95% CI 50.3–58.3%, *P* < 0.001) of their pain (Table 4). Additionally, cannabis users estimated that 61.3% ± 4.6 (95% CI 56.7–65.9%, *P* < 0.001) of their pain can be treated by cannabis, while non-cannabis users believed this to be 49.1 ± 7.5 (95% CI 41.6 to 56.6, *P* < 0.001). Patients estimated 48.9% ± 7.3 (95% CI 41.6–56.1%, *P* < 0.001) of their current pain medication regimens consisted of cannabis and believed cannabis could replace 46.2% ± 6. 6 (95% CI 39.6–52.8%) of their opioid analgesics. Approximately a quarter of the patients (27.9%, 65/233) answered correctly that cannabidiol (CBD) was responsible for the pain-relieving effects, and 44.4% (104/234) recognized that the psychotropic effects come from tetrahydrocannabidiol (THC). Most patients felt comfortable discussing cannabis use with their physicians 79.7% ± 4.3 (95% CI 75.4 -84.0%) (Table 4).

**Table 4:** Perceptions regarding cannabis use following MSK injury

Variable	Mean value (95%CI)
Percentage of pain that cannabis can/could treat (0% = none, 100% = all)	54.3% (50.3–58.3%)
Percentage of pain that cannabis can/could treat for cannabis users (0% = none, 100% = all)	61.3% (56.7–65.9%)
Percentage of pain that cannabis can/could treat for non-cannabis users (0% = none, 100% = all)	49.1% (41.6–56.6%)
Percentage of their pain medication regime is made up by cannabis (0% = none, 100% = all)	48.9% (41.6–56.1%)
Percentage of analgesic medications that cannabis does/could replace (0% = none, 100% = all)	46.2% (39.6–52.8%)
Comfort in discussing cannabis use with provider (0% = not comfortable at all, 100% = completely comfortable)	79.7% (75.4–84.0%)

Age (*β* = − 0.3, CI − 0.6–0.0, P = 0.033) and patients reporting higher pain severity on the VAS were associated with reporting a higher perceived effect of cannabis (*β* = 0.4, CI 0.1–0.6, *P* = 0.005) (Table 5). Additionally, patients who previously used cannabis were also more likely to report a higher perceived effect of cannabis (*β* = 14.7, CI 5.1–24.4, *P* = 0.003).

**Table 5:** Multivariable regression model for patients’ perception of their pain treated by cannabis

Covariate	*ß* coefficient	95% CI	*P*-value
Age	− 0.3	− 0.6–0.0	0.033*
*Sex*			
Male	− 6.1	− 16.2–4.1	0.237
Female	–	–	–
*Duration of pain/symptoms*			
Less than 6 months (acute)	–	–	–
6 months or more (chronic)	11.0	− 1.9–24.0	0.094
Had surgery	3.6	− 6.0–13.1	0.459
Pain severity (VAS)	0.4	0.1–0.6	0.005*
Used cannabis to manage pain in last year	14.7	5.1–24.4	0.003*
Anxiety	− 0.1	− 10.8–10.6	0.981
PTSD	− 5.2	− 15.9–5.6	0.343

*p-value  < 0.05

### Knowledge, attitudes, and preferences regarding cannabis

**Fig. 1:**
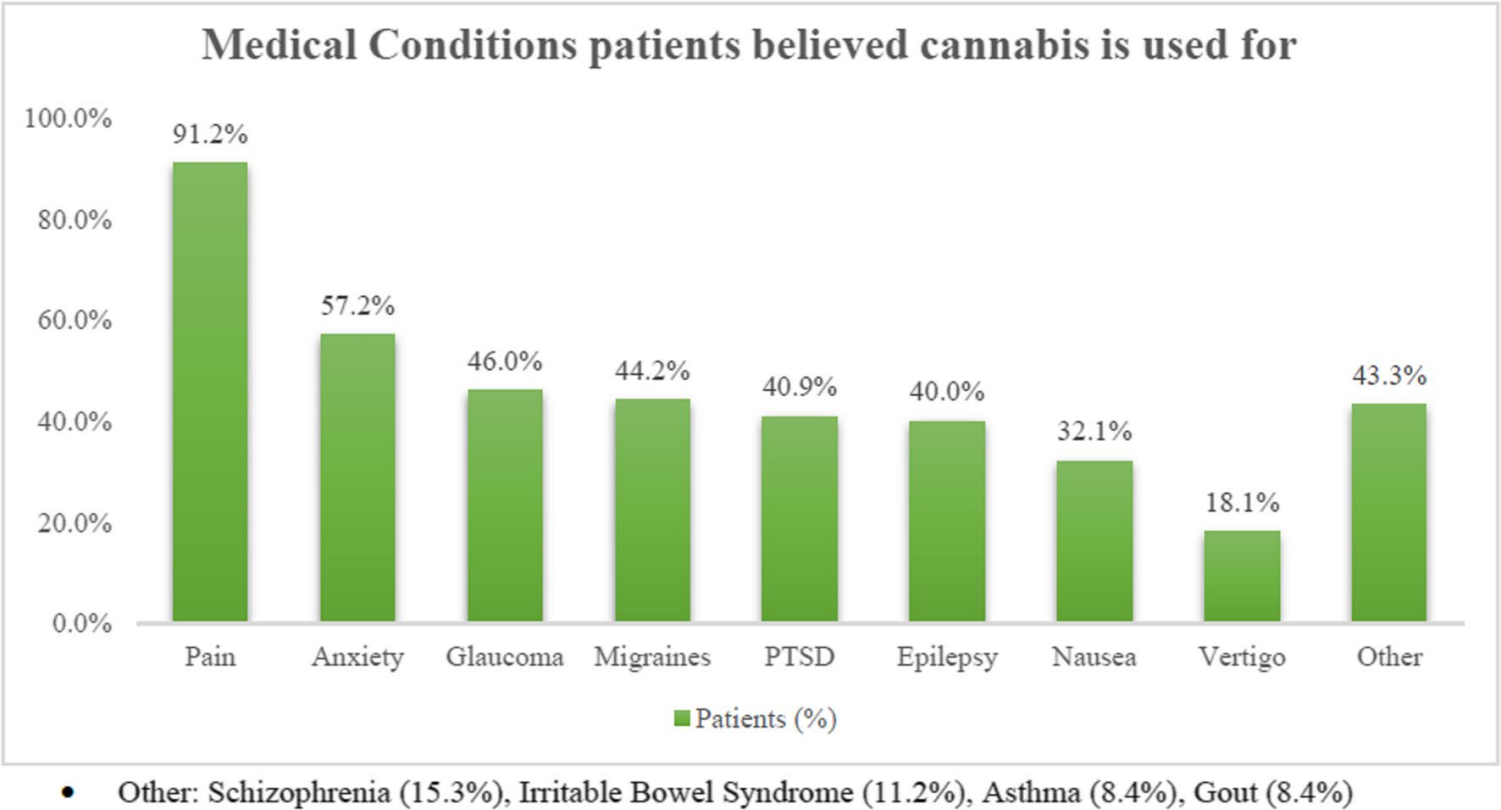
Medical conditions patients believed cannabis is used for

The patients recognized anxiety (57.2%, 123/215), migraines (44.2%, 95/215), PTSD (40.9%, 88/215), glaucoma (46.0%, 99/215), nausea (32.1%, 69/215), and epilepsy (40.0%, 86/215) as indications for cannabis. (Fig. 1). However, the majority of patients (91.2%, 126/215) also believed cannabis was already approved for treating pain, which it was not.

**Fig. 2:**
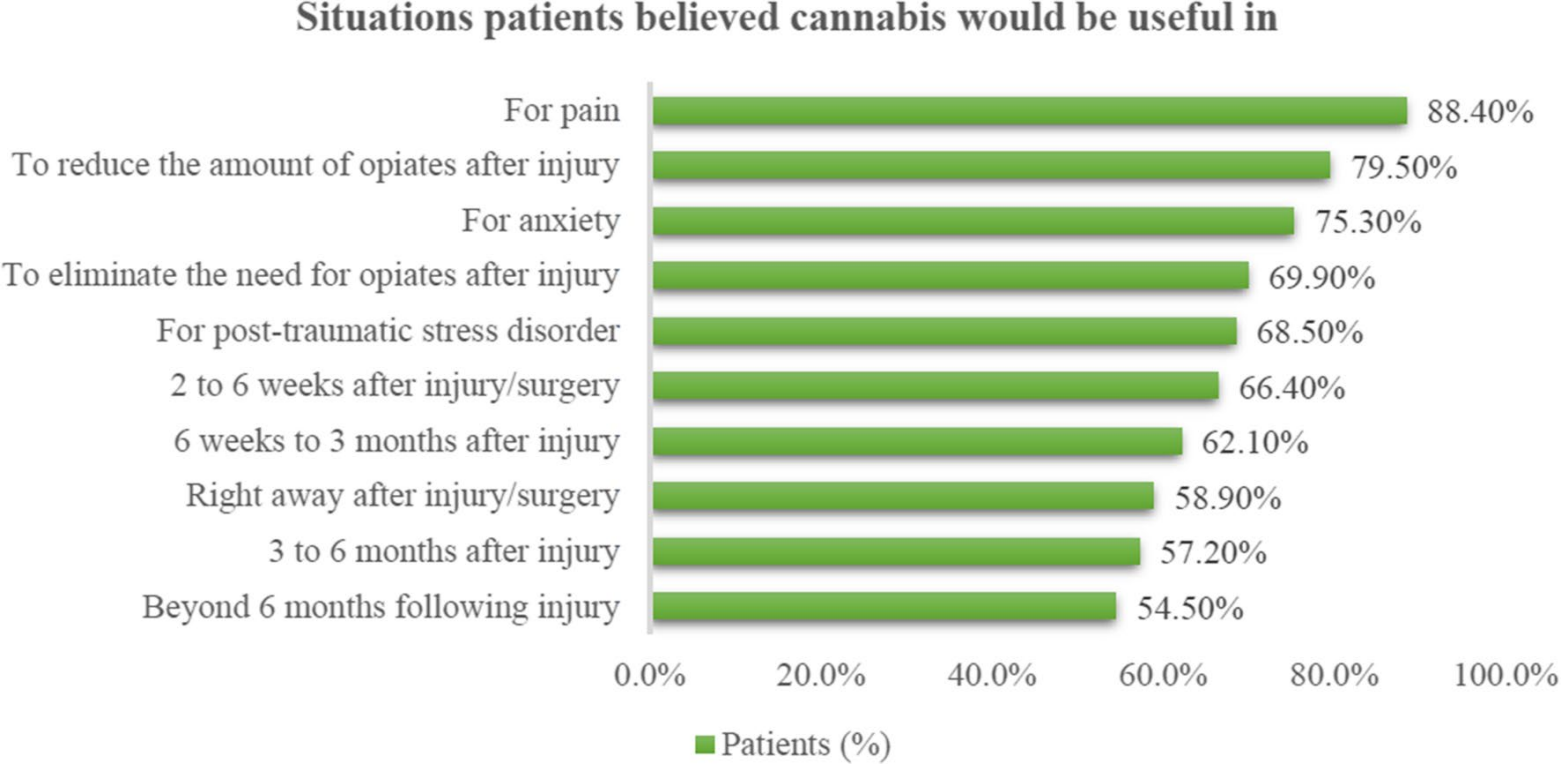
Situations patients believed cannabis would be useful in

Timing-wise, the patients felt that between 2 and 6 weeks (66.4%, 97/146) and between 6 weeks and 3 months (62.1%, 90/146) was the most appropriate period to use cannabis (Fig. 2). Additionally, the majority thought that using cannabis at multiple time points could also be beneficial (from immediately afterward to beyond 6-months). Regarding specific situations, patients believed cannabis could be useful for treating pain (88.4%, 129/146), decreasing opioid use after injury (79.5%, 116/146), anxiety (75.3%, 110/146), and PTSD (68.5%, 100/146).

Regarding barriers, patients reported not considering using cannabis for medical purposes (43.3%, 65/150), as the most common reason for not discussing cannabis with physicians (Table 6). Only 30.8% (73/237) of the patients reported discussing cannabis for back pain with their physicians. Of those who did discuss it, 64.8% (46/71) described it as a positive experience. More than half of patients (50.4%, 117/232) of patients stated they would participate in a randomized trial on cannabis for pain relief. (Table 6).

**Table 6:** Barriers and considerations for clinical use and further investigation

Variable	No. of patients (%)	
*Have discussed medical use of cannabis with physician (N = 237)*		
*No*	164 (69.2%)	
*Yes*	73 (30.8%)	
I never thought about using cannabis for medical purposes*	65 (43.3%)	
I am concerned about addiction with cannabis*	27 (18.0%)	
I don’t need any more medications for pain control*	23 (15.3%)	
I am concerned about side effects*	22 (14.7%)	
I can easily obtain cannabis through other physicians/sources*	15 (10.0%)	
I do not know how to access cannabis*	15 (10.0%)	
I don’t think it works*	14 (9.3%)	
My physician doesn’t think it works*	11 (7.3%)	
I have a moral or religious objection to using cannabis*	6 (4.0%)	
Other*	18 (12.0%)	
*Was the discussion a positive experience (N = 71)*		
*Very positive*	31 (43.7%)	
*Positive*	15 (21.1%)	
*Mixed*	14 (19.7%)	
*Somewhat negative*	9 (12.7%)	
*Very negative*	2 (2.8%)	
Where patients obtained/preferred to obtain cannabis from	Current (*N* = 81)	Preferred (*N* = 79)
*Government*	28 (34.6%)	45 (57.0%)
*Private dispensary*	37 (45.7%)	35 (44.3%)
*Online*	35 (43.2%)	35 (44.3%)
*Home grown*	11 (13.6%)	22 (27.9%)
*Forms of cannabis patients preferred to use to treat their pain (N = 235)*		
*Oral pill/tablet*	98 (41.7%)	
*Edible*	67 (28.5%)	
*Sublingual*	58 (24.7%)	
*Inhaled smoke*	48 (20.4%)	
*Topical*	44 (18.7%)	
*Inhaled vapor*	44 (18.7%)	
*Liquid*	40 (17.0%)	
*Transdermal*	23 (9.8%)	
*Intra-articular*	8 (3.4%)	
*Willingness to participate in a randomized clinical trial comparing cannabis to usual care for pain relief following an MSK injury*		
*Yes*	117 (50.4%)	
*No*	51 (22.0%)	
*Unsure*	64 (27.6%)	

*MSK* Musculoskeletal

*denominator is 150, as 150 patients responded to that question

Concerns about the side effects of cannabis use and addiction were expressed by 14.0% (21/150) and 18% (27/150) of patients, respectively (Table 6). Of the people who had obtained cannabis previously, most commonly reported method for obtaining cannabis were private dispensaries (45.7%, 37/81) and online (43.2%, 35/81). However, in general, patients would prefer to get their cannabis through government-licensed producers (57.0%, 45/79) (Table 6). Patients reported that they would prefer oral (41.7%, 98/235) or edible formulations (28.5%, 67/235).

## Discussion

The opioid crisis in North America is worsening. Deaths involving opioids increased by 500% in 2017 compared to 2016, and the COVID-19 pandemic further exacerbated this public health problem by adding more strain on the healthcare system [16, 19, 27]. Opioid prescriptions play a substantial role in these deaths and are still commonly prescribed for MSK pain. Finding a safe and effective non-opioid alternative is critical.

This survey asked 259 patients presenting to Orthopedic clinics about their beliefs and perceptions regarding the effect of cannabis on spine pain. The literature on cannabis for MSK pain is somewhat inconsistent [28]. The National Academies for Science, Engineering, and Medicine of the USA published a review stating that cannabis was effective for non-cancer chronic pain treatment [29]. However, a year later, a systematic review concluded that the evidence for the effectiveness of medical cannabis on chronic non-cancer pain is limited [30]. Our findings show that 88.4% of the patients believed cannabis was an effective treatment option for back pain and could treat 54.3% of their pain and replace nearly half (46.2%) of their current pain medications. Additionally, most patients believed cannabis would be more beneficial for treating their pain if started earlier, specifically in the first three months. A third (35.3%) already used medical cannabis for treating pain, most within the last year.

Nearly three-quarters of the patients in this study used opioids to manage their back pain. Considering all the campaigns aimed at restricting opioid use for chronic non-cancer pain, these findings are very concerning and seem to be a common theme, as other studies have also shown opioids being used as a first-line for MSK pain [7, 31–33]. However, in our study, eight out of ten patients also felt cannabis could decrease their opioid needs. A small qualitative study including 20 patients with MSK pain showed similar findings, and although not specific to MSK and spine pain, there is other data that endorses the potential of cannabis to reduce opioid use [34–37]. This finding further supports considering cannabis for musculoskeletal pain control.

Patients generally felt comfortable discussing cannabis with their physicians, with nearly two-thirds (64.8%) describing it as a positive experience. However, only a third (30.8%) reported having had a conversation with their physicians on cannabis for their pain. The number one barrier patients stated was not knowing they could use cannabis for medical purposes. Considering that concerns regarding side-effects or addiction were low (14–18%), we believe this is likely due to the fact that cannabis is not yet approved for treating MSK pain by most regulatory bodies, such as Health Canada or the Food and Drug Administration (FDA).

As this was a survey-based study, the main limitation was recall bias. Nonresponse bias could also be considered a limitation, as cannabis is still considered a schedule 1 drug in the US and associated with some stigma [38, 39]. Hopefully, the recent shift toward more permissive views on cannabis and our study showing that moral or religious objections to using cannabis were low (4%) has helped offset this inclination. A potential weakness could be considered that the study was done in 2018; however, in Canada, legislation regarding medical cannabis has not changed since then, and the change in the legal status of recreational cannabis was in 2018, which is when we conducted the study.

There is an increased interest in exploring the potential analgesic applications of cannabis [40]. There has been evidence showing that cannabis can be effective for back pain, although the quality of the evidence is poor [21, 41]. In our study, patients reported interest in participating in randomized clinical trials (RCT) comparing cannabis to standard treatments, as more than half of the participants (54.8%) responded they would participate in such a clinical trial.

Patients with spine pain perceived cannabis as an effective pain medication, with one in three already using it for their back pain. Additionally, cannabis could potentially be used for opioid-sparing purposes, as eight out of ten patients also believed cannabis could reduce opioid use and replace nearly half of their opioid medications. Before we can add cannabis as another resource to our pain management arsenal, more research and high-quality data from RCTs are necessary. Our data on the barriers (educating physicians and patients on cannabis), timing (acute period), and preferences (government distributors and oral formulations) could help guide future research and clinical application of cannabis.

## Data Availability

The datasets used and/or analyzed during the current study are available from the corresponding author on reasonable request.

## Abbreviations

US: United States
NSAIDs: Nonsteroidal anti-inflammatory drugs
MSK: Musculoskeletal
ON: Ontario
VAS: Visual analog scale
PTSD: Post-traumatic stress disorder
PROMIS: Patient-Reported Outcomes Measurement Information System
CBD: Cannabidiol
RCT: Randomized clinical trials
FDA: Food and Drug Administration
